# Identification of Genes with Rare Loss of Function Variants Associated with Aggressive Prostate Cancer and Survival

**DOI:** 10.1016/j.euo.2024.02.003

**Published:** 2024-03-07

**Authors:** Edward J. Saunders, Tokhir Dadaev, Mark N. Brook, Sarah Wakerell, Koveela Govindasami, Reshma Rageevakumar, Nafisa Hussain, Andrea Osborne, Diana Keating, Artitaya Lophatananon, Kenneth R. Muir, Burcu F. Darst, David V. Conti, Christopher A. Haiman, Antonis C. Antoniou, Rosalind A. Eeles, Zsofia Kote-Jarai

**Affiliations:** a Division of Genetics and Epidemiology, The Institute of Cancer Research, London, UK;; b Division of Population Health, University of Manchester, Manchester, UK;; c Center for Genetic Epidemiology, Department of Population and Public Health Sciences, University of Southern California, Los Angeles, CA, USA;; d Public Health Sciences, Fred Hutchinson Cancer Center, Seattle, WA, USA;; e Department of Public Health and Primary Care, University of Cambridge, Cambridge, UK;; f The Royal Marsden NHS Foundation Trust, London, UK

**Keywords:** Aggressive prostate cancer, DNA repair genes, Germline prognostic markers, Prostate cancer, Prostate cancer survival

## Abstract

**Background:**

Prostate cancer (PrCa) is a substantial cause of mortality among men globally. Rare germline mutations in *BRCA2* have been validated robustly as increasing risk of aggressive forms with a poorer prognosis; however, evidence remains less definitive for other genes.

**Objective:**

To detect genes associated with PrCa aggressiveness, through a pooled analysis of rare variant sequencing data from six previously reported studies in the UK Genetic Prostate Cancer Study (UKGPCS).

**Design, setting, and participants:**

We accumulated a cohort of 6805 PrCa cases, in which a set of ten candidate genes had been sequenced in all samples.

**Outcome measurements and statistical analysis:**

We examined the association between rare putative loss of function (pLOF) variants in each gene and aggressive classification (defined as any of death from PrCa, metastatic disease, stage T4, or both stage T3 and Gleason score ≥8). Secondary analyses examined staging phenotypes individually. Cox proportional hazards modelling and Kaplan-Meier survival analyses were used to further examine the relationship between mutation status and survival.

**Results and limitations:**

We observed associations between PrCa aggressiveness and pLOF mutations in *ATM*, *BRCA2*, *MSH2*, and *NBN* (odds ratio = 2.67–18.9). These four genes and *MLH1* were additionally associated with one or more secondary analysis phenotype. Carriers of germline mutations in these genes experienced shorter PrCa-specific survival (hazard ratio = 2.15, 95% confidence interval 1.79–2.59, *p* = 4 × 10^−16^) than noncarriers.

**Conclusions:**

This study provides further support that rare pLOF variants in specific genes are likely to increase aggressive PrCa risk and may help define the panel of informative genes for screening and treatment considerations.

**Patient summary:**

By combining data from several previous studies, we have been able to enhance knowledge regarding genes in which inherited mutations would be expected to increase the risk of more aggressive PrCa. This may, in the future, aid in the identification of men at an elevated risk of dying from PrCa.

## 1. Introduction

Prostate cancer (PrCa) is the most frequently diagnosed cancer site and second highest cancer-related cause of mortality among males in the UK [1], as well as a substantial cause of mortality globally [2]. Although an appreciable subset of PrCa patients develops aggressive forms with a poorer prognosis, the majority experience indolent, slow developing disease that may not substantively reduce their length or quality of life [3,4].

PrCa demonstrates a high level of heritability [5,6], including evidence for concordance of more or less favourable outcomes within families [7]. In recent years, multiple common, low-penetrance genetic loci have been identified, which cumulatively exert substantial influence upon PrCa incidence [8,9]. Genetic risk scores (GRS) based on these common PrCa risk variants have, however, thus far demonstrated limited or currently uncertain direct capability towards prognostic discrimination [10–12]. A small number of genes have also been identified in which rare, moderate penetrance mutations confer greater effects upon PrCa risk, with low-frequency recurrent variants in *HOXB13* robustly associated with a greater risk of PrCa of any severity [13–15] and rare germline mutations in *BRCA2* associated with a greater risk of aggressive PrCa [16–21]. Associations with aggressive PrCa have also been reported for *ATM* [18,21], *NBN* [21,22], and *PALB2* [18], although further replication remains warranted. Germline mutations in additional candidate genes, particularly DNA repair genes, have also been observed at low frequencies among patients with metastatic PrCa; however, the majority of these studies were underpowered to evaluate the strength of evidence in support of an association with disease aggressiveness for specific genes [7].

In this study, we have aggregated the existing germline rare variant sequencing data for PrCa cases from the UK that were collected as part of the UK Genetic Prostate Cancer Study (UKGPCS) [23] and sequenced as part of six prior datasets [18,19,21,24–26], to conduct a larger pooled analysis. In total, we evaluated a panel of ten commonly proposed candidate genes for aggressive PrCa in 6805 PrCa cases, including 3548 with aggressive PrCa. We also investigated the combined effects of rare and common variation on PrCa aggressiveness, to assess whether common variation associated with PrCa onset could exacerbate or mitigate the likelihood of experiencing clinically significant disease conferred by rare moderate penetrance variants linked to poorer outcomes.

## 2. Patients and methods

### 2.1. Study sample

Data from European ancestry samples consented into the UKGPCS as part of six separate retrospective whole-exome or gene panel sequencing studies were included in this pooled analysis. The individual studies had differing sample selection criteria, sample sizes, and gene panel compositions (Supplementary Table 1), with three studies selecting for cases with aggressive or nonaggressive clinical presentation [18,21,25], two for cases with a family history (FH) of PrCa [24,26], and one for PrCa patients with an age at diagnosis of <65 yr [19]. For nonunique samples, duplicates for retention were prioritised from studies with larger sample sizes and numbers of genes sequenced. As the constituent studies for this pooled analysis had differing PrCa case inclusion criteria but broadly employed extreme phenotype approaches, the final dataset available for analysis was enriched for patients with aggressive, fatal, and younger onset disease, in addition to an FH of PrCa (Table 1).

### 2.2. Variant categorisation

We curated a set of ten genes (*ATM*, *BRCA1*, *BRCA2*, *CHEK2*, *MLH1*, *MSH2*, *MSH6*, *MUTYH*, *NBN*, and *PALB2*) that had been sequenced in all samples available for the pooled analysis and are regularly identified as candidates for associations with PrCa risk and disease aggressiveness. Variants within these genes were annotated with the variant effect predictor (VEP) [27]. Only rare putative loss of function (pLOF) variants were included in downstream analyses. Specifically, variants were retained that had minor allele frequency (MAF) <0.01 in each ancestral reference population from 1000 genomes, ESP, or gnomAD (VEP “max_af” <0.01), and either were protein truncating (VEP impact = high and LOFTEE LoF = high confidence) or if not were classified as “pathogenic” or “likely pathogenic” in ClinVar (nontruncating variants with conflicting interpretations of pathogenicity were excluded) [28].

### 2.3. Genetic risk score calculation

We calculated a GRS for the risk of PrCa incidence for UKGPCS samples using imputed genotype data generated previously as part of the OncoArray genotyping array [29]. In total, data were available for 444 out of 451 established PrCa risk variants [9] for 5606 of the 6805 UKGPCS samples in the pooled analysis (82%). An additional 393 variant GRS was also considered, which excluded 51 variants associated with prostate-specific antigen (PSA) levels [30].

### 2.4. Statistical analysis

Individual genes were assessed for an association with aggressive PrCa phenotypes using Firth logistic regression models adjusted for study (six-level categorical variable) and continuous age at PrCa diagnosis. Chi-square tests for trend were additionally used to investigate the trends in the Gleason grade group and tumour stage variables. Owing to the rarity of individual pLOF variants, mutation status was defined as a binary variable, indicating the presence of one or more variants in the gene or gene set analysed. Odds ratios (OR) and 95% confidence intervals (95% CI) were estimated for the association between mutation status and the phenotype under consideration.

The primary analysis examined the association between rare variants in each gene and aggressive PrCa, adopting an omnibus criterion to describe aggressive PrCa presentation. Cases were defined as “aggressive” if they had at least one of the following: cause of death recorded as PrCa, metastatic disease, stage T4, and both stage T3 plus Gleason score ≥8. Cases were defined as “nonaggressive” if they had all of the following: stage ≤T2, Gleason score ≤6, and if deceased, death was not due to PrCa. Cases classified as neither aggressive nor nonaggressive (ie, those without metastatic spread, who had not died of PrCa, and who had Gleason score ≥8 plus stage ≤T2, Gleason score 7 plus stage ≤T3, or Gleason score ≤6 and stage T3) were regarded as intermediate aggressiveness and excluded from the primary comparison of aggressive versus nonaggressive PrCa, but were available for inclusion in secondary analyses examining the association with individual phenotypic indicators of aggressiveness. Secondary analyses assessed the associations with metastases, nodal spread, Gleason grade group (≤2 vs ≥3), and T stage (≤2 vs ≥3). Samples lacking data for any criterion were excluded from analyses of the relevant phenotype. We report associations between any gene and phenotype significant at *p* < 0.05 and additionally indicate associations significant at a *p* < 0.001 threshold, representing a conservative adjustment accounting for multiple testing of ten genes against five phenotypes across the primary and secondary analyses under the assumption of independence between tests.

Delayed entry Cox proportional hazard regression models, with time since diagnosis as the timescale, were used to calculate hazard ratios (HR) and 95% CI for the association between mutation carrier status and each of PrCa-specific, all-cause, and non-PrCa mortality. Survival probability stratified by mutation status was visualised using Kaplan-Meier plots. Genes associated with aggressive PrCa in the Firth logistic regression analysis defined the “gene set”, with individuals having a mutation in any gene among the gene set classified as “carriers”. Individuals became at risk at their age at PrCa diagnosis and under observation at their date of consent to the UKGPCS, with the time to event calculated from age at diagnosis to death. Patients who did not die were censored at their age of last follow-up in the all-cause analysis, and patients additionally censored at their age of death from other causes in the PrCa-specific analyses or age at death from PrCa in the non-PrCa mortality analyses. We used competing-risks regression to account for other-cause mortality in the PrCa-specific analysis. Individuals who were still alive at emigration or on June 28^th^ 2019 were censored at the earlier of these dates; this date was chosen because it is the latest date where mortality flagging information is known to be complete. We excluded from this analysis 17 participants who could not be traced under the Medical Research Information Service/NHS Digital and 117 participants from Northern Ireland for whom tracing data were not complete. We estimated the total effect of mutation status on survival without adjustment for clinical variables under the hypothesis that both are factors on the causal pathway towards aggressive disease, adjusting for age at diagnosis (linear trend), study (six-level categorical variable), and year of diagnosis (<2000, 2000–2004, 2005–2009, and ≥2010), although additionally report the direct effect of mutation status on mortality after also adjusting for clinical variables for comparative purposes.

Association tests between GRS for common PrCa risk variants and mutation status in the gene set associated with aggressive PrCa were performed for aggressive classification, adjusting for study and age at diagnosis, using Firth logistic regression with GRS as a continuous variable. Additional tests for interaction between GRS and mutation status with aggressive classification were also conducted to further evaluate the combined influence of common variants associated with PrCa incidence and rare variants associated with PrCa aggressiveness upon clinically relevant characteristics in PrCa cases.

All analyses were performed using R (version 4.3.1) and Stata (18.0; StataCorp LLC, College Station, TX, USA).

## 3. Results

In total, rare variant information for 8183 PrCa cases was available, with data from 6805 unique individuals remaining for analysis after the exclusion of interstudy duplicates and relatives. In the final sample set, 539 (7.9%) individuals were carriers of a rare pLOF variant in one or more of the ten candidate genes examined, with 551 pLOF variants identified in total (Supplementary Tables 2 and 3). The most frequently mutated genes in the dataset were *MUTYH* (*n* = 136, 2.0%), *BRCA2* (*n* = 118; 1.7%), *CHEK2* (*n* = 95, 1.4%), and *ATM* (*n* = 91; 1.3%). pLOF variants were present at lower rates in the remaining genes, with *MSH6* being the next most frequently mutated (*n* = 36, 0.53%) and *MLH1* containing the fewest (*n* = 11, 0.16%). Ten individuals were carriers of a mutation in two genes and one individual in three genes, nine of whom were classified to have aggressive disease, seven had died from PrCa (median survival duration 4 yr, range 2–6 yr), and two had a known FH of PrCa (Supplementary Table 4).

**Fig. 1 –:**
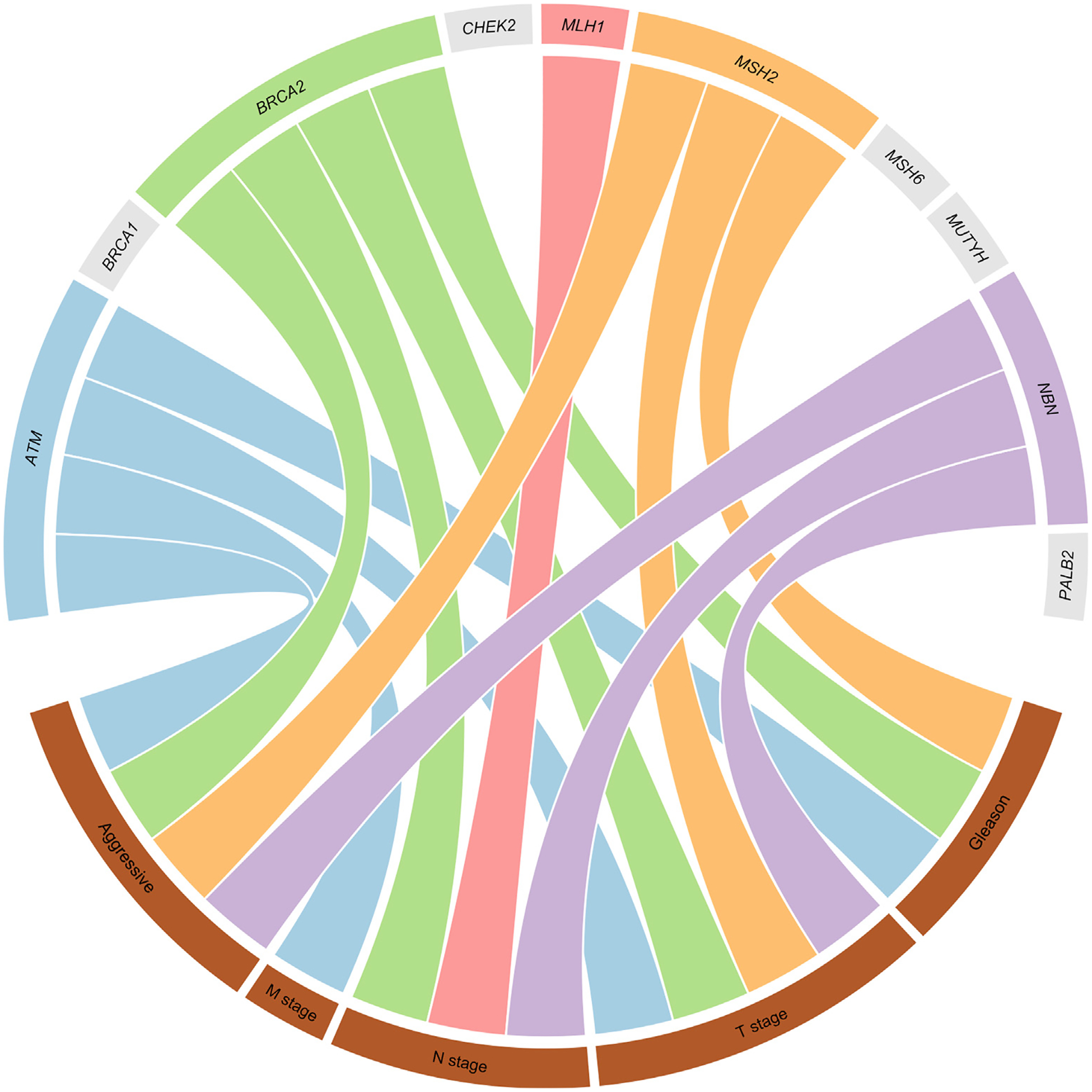
Flow diagram depicting significant associations between genes and phenotypes in the primary and secondary analyses. Genes are shown on the top and phenotypes at the bottom, with links indicating association. Genes for which no association was observed for any phenotype are shown as grey segments.

We observed associations between rare pLOF variants in five genes and aggressive PrCa phenotypes (Fig. 1). In the primary analysis of aggressive PrCa classification, there were significant differences between aggressive and nonaggressive disease for *ATM* (OR = 2.67, 95% CI 1.56–4.56, *p* < 0.001), *BRCA2* (OR = 5.13, 95% CI 2.98–8.83, *p* < 0.001), *MSH2* (OR = 5.17, 95% CI 0.93–28.7), and *NBN* (OR = 18.9, 95% CI 1.12–320) mutation carriers (Supplementary Table 5). In the secondary analyses (Supplementary Table 6), *ATM* pLOF variants were associated with metastatic spread (OR = 2.15, 95% CI 1.28–3.60), and pLOF variants in *BRCA2* (OR = 1.83, 95% CI 1.13–2.95), *MLH1* (OR = 5.89, 95% CI 1.14–30.4), and *NBN* (OR = 3.67, 95% CI 1.12–12.1) were associated with nodal invasion. *ATM* (OR = 2.24, 95% CI 1.42–3.52, *p* < 0.001), *BRCA2* (OR = 2.40, 95% CI 1.59–3.62, *p* < 0.001), *MSH2* (OR = 12.5, 95% CI 2.26–68.9, *p* < 0.001) and *NBN* (OR = 3.77, 95% CI 1.10–12.8) pLOF mutations were also associated with higher T stage, and *ATM* (OR = 2.11, 95% CI 1.34–3.30), *BRCA2* (OR = 3.57, 95% CI 2.35–5.42, *p* < 0.001) and *MSH2* (OR = 5.03, 95% CI 1.45–17.4) pLOF mutations were associated with higher Gleason score.

Although pLOF *MLH1* mutations were associated with nodal spread only and not the primary aggressiveness criteria, *MLH1* mutations were also observed four-fold more often in cases with metastases than without, twice as frequently in cases with T stage ≥3 than T stage ≤2, and three times more often among cases with Gleason grade group ≥3 than group 2. Among the genes that were not significantly associated with any phenotype in the primary and secondary analyses, two-fold or greater elevated pLOF mutation carrier frequencies were observed relative to the more favourable outcome group for *BRCA1* in patients with metastases, T stage ≥3, and Gleason grade group ≥3, and for *PALB2* in patients with nodal spread and Gleason grade group ≥3 (Supplementary Table 6 and Supplementary Fig. 1). Carrier frequencies of rare pLOF mutations in *ATM*, *BRCA1*, *BRCA2*, *MLH1*, *MSH2*, *NBN*, and *PALB2* were all elevated in patients who died of PrCa relative to those who had not died (Supplementary Table 7 and Supplementary Fig. 1).

When considering T stage and Gleason grade group as categorical rather than dichotomised variables, higher cumulative pLOF mutation carrier frequencies for the five genes associated with aggressive PrCa phenotypes (*ATM*, *BRCA2*, *MLH1*, *MSH2*, and *NBN*) were observed with both increasing tumour stage (*P-trend* = 3.9 × 10^−12^) and Gleason grade group (*P-trend* = 9.6 × 10^−15^), with the carrier frequencies in patients with T4 tumour stage or grade group 5 Gleason pattern being approximately three and a half times those with T1 or grade group 1 phenotypes (Supplementary Table 8 and Supplementary Fig. 2). In a logistic regression analysis adjusted for study, mutation status in the five genes associated with aggressive PrCa was weakly correlated with a positive FH of PrCa, although this association was not significant (OR = 1.27, 95% CI 0.95–1.68, *p* = 0.1). We also investigated the combined effect of a GRS developed for the prediction of PrCa incidence alongside rare pLOF mutations in the five genes that demonstrated differences between aggressive and nonaggressive disease. In a multivariable model looking at the association between aggressive classification with GRS and mutation status, aggressive disease was strongly positively associated with mutation status (OR = 2.02, 95% CI 1.59–2.56, *p* = 8.1 × 10^−9^) and weakly negatively associated with GRS (OR per SD = 0.93, 95% CI 0.87–0.99, *p* = 0.03). There was no suggestion of interaction between GRS and mutation status (Supplementary Table 9). The association between aggressive disease and GRS became nonsignificant and in the positive direction upon the exclusion of 51 variants that have also been reported to associate with PSA levels (OR = 1.05, 95% CI 0.98–1.13, *p* = 0.2).

**Fig. 2 –:**
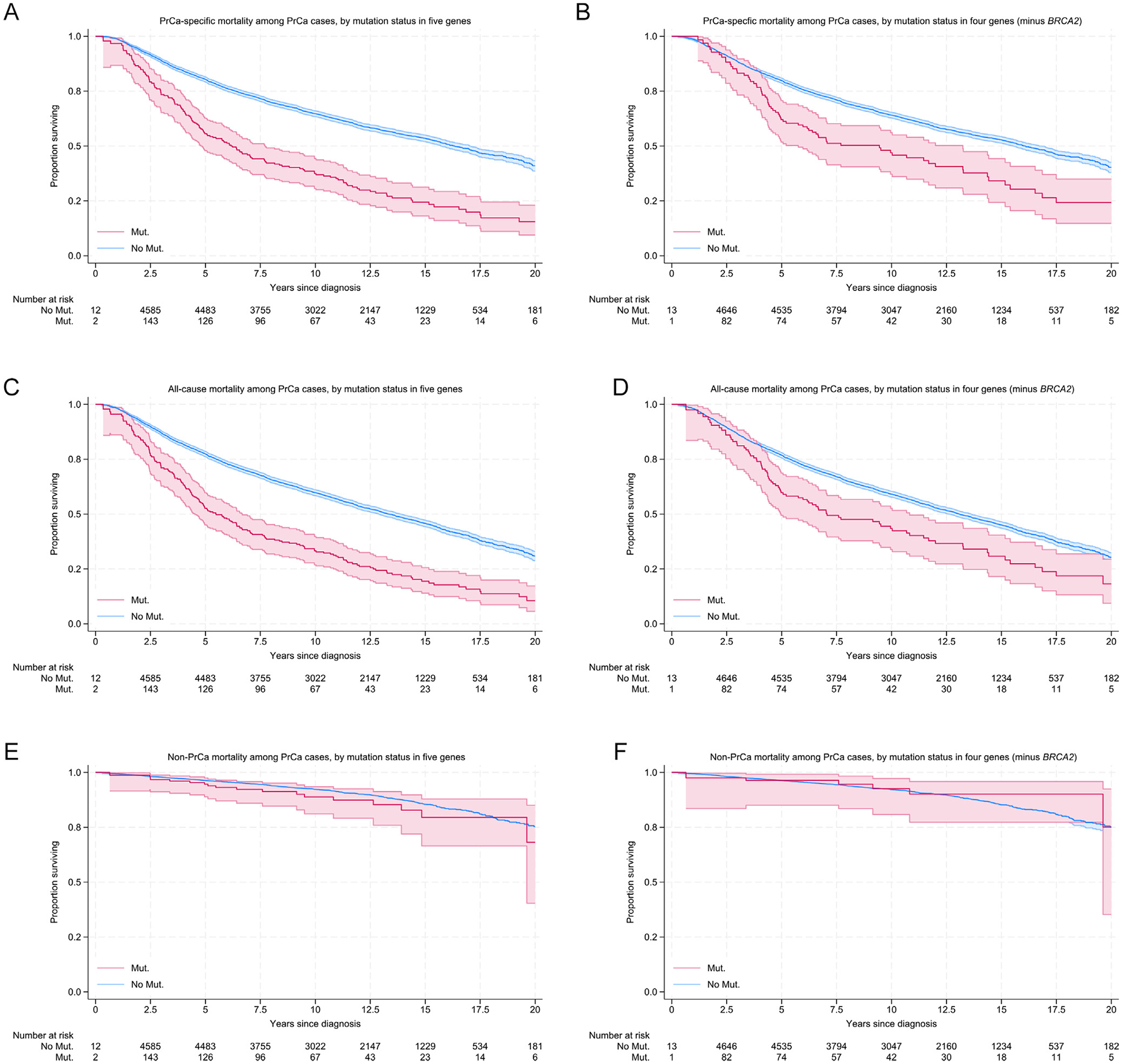
Kaplan-Meier plots depicting relative survival probability against time in years between mutation carriers for the five genes associated with aggressive PrCa (*ATM*, *BRCA2*, *MLH1*, *MSH2*, and *NBN*) and noncarriers, and with *BRCA2* excluded: (A) PrCa-specific death for the set of five genes associated with aggressiveness, (B) PrCa-specific death with *BRCA2* excluded from the gene set, (C) all causes of death for the set of five genes associated with aggressiveness, (D) all causes of death with *BRCA2* excluded from the gene set, (E) non-PrCa cause of death for the set of five genes associated with aggressiveness, and (F) non-PrCa cause of death with *BRCA2* excluded from the gene set. Mut. = mutation; PrCa = prostate cancer.

In time-to-event analyses, we assessed the association between mutation status in any of the five genes associated with aggressive PrCa phenotypes and survival. As the gene set was defined through an association with PrCa aggressiveness and PrCa aggressiveness is directly related to poorer survival, we hypothesise that both mutation status and clinical variables would represent factors on the causal pathway to fatal disease, whereby the clinical features serve as mediators of effects conferred by genetic variants associated with aggressiveness, rather than confounders. Accordingly, we therefore primarily examined the total effect of mutation status upon survival, although also report the direct effect after adjustment for clinical variables to enable evaluation of the alternative scenario. The median follow-up duration was 10.4 yr, with 82% of samples having ≥5 yr of follow-up. Carriers of rare pLOF mutations in these genes experience shorter PrCa-specific survival (HR = 2.15, 95% CI 1.79–2.59, *p* = 4 × 10^−16^) than noncarriers (Fig. 2 and Table 2). This association remained significant although was somewhat attenuated when excluding *BRCA2* from the list of genes (*BRCA2*-excluded HR = 1.78, 95% CI 1.38–2.30, *p* = 9 × 10^−6^; *BRCA2*-only HR = 2.56, 95% CI 1.98–3.31, *p* = 1 × 10^−12^). Similar results were observed for all-cause mortality (HR = 2.15, 95% CI 1.84–2.52, *p* = 2 × 10^−21^; *BRCA2*-excluded HR = 1.59, 95% CI 1.27–2.00, *p* = 5 × 10^−5^; and *BRCA2*-only HR = 2.96, 95% CI 2.39–3.66, *p* = 2 × 10^−23^), although the majority (81%) of deaths recorded in the sample set were due to PrCa. Whilst *MLH1* had been associated with nodal spread only and not the broader aggressiveness criteria, exclusion of *MLH1* from the gene set did not demonstrate a meaningful difference in mortality (*MLH1*-excluded PrCa-specific mortality HR = 2.18, 95% CI 1.80–2.62, *p* = 4 × 10^−16^; all-cause mortality HR = 2.17, 95%CI 1.85–2.55, *p* = 4 × 10^−21^). For the subset of PrCa cases recorded as having died from non–PrCa-related causes, the difference in survival time between carriers of rare pLOF mutations and noncarriers was reduced and not significant (HR = 1.17, 95% CI 0.74–1.85, *p* = 0.5), but poorer other-cause survival was observed for *BRCA2* carriers in contrast to carriers of mutations in the other four genes (*BRCA2*-excluded HR = 0.73, 95% CI 0.36–1.50, *p* = 0.4; *BRCA2*-only HR = 1.75, 95% CI 0.97–3.17, *p* = 0.06). The estimated direct effect of mutation status for the five genes associated with aggressive PrCa on mortality when adjusting for clinical variables was substantially lower than the total effect, although remained significantly associated (PrCa-specific mortality HR = 1.27, 95% CI 1.04–1.55, *p* = 0.02; all-cause mortality HR = 1.29, 95% CI 1.10–1.51, *p* = 0.002).

## 4. Discussion

Early detection and treatment of clinically significant prostate tumours, and avoidance of overtreatment of slow progressing forms represent competing considerations for prospective screening and treatment approaches for PrCa. Whilst this could potentially be mitigated by genetic information, the discovery of genes in which pathogenic mutations predispose towards a higher likelihood of developing aggressive PrCa is hindered by low carrier rates of mutations in individual genes and incomplete penetrance. In this analysis, we pooled germline sequencing data previously accrued for PrCa cases from a single UK-based study and examined ten genes frequently reported as candidates sequenced in all constituent datasets.

We were able to identify associations between rare pLOF mutations in *ATM*, *BRCA2*, *MSH2*, and *NBN*, and aggressive classification, in addition to further associations between these four genes, plus *MLH1*, with individual clinical phenotypes indicative of aggressiveness. Our findings further support those of a recent large multicountry European ancestry study of aggressive PrCa, comprising 9185 aggressive and 8361 nonaggressive PrCa cases from Australia, the USA, the UK, Finland, Sweden, and other European countries, which reported exome-wide significant associations between *ATM* and *BRCA2* mutations and aggressive PrCa classification, alongside suggestive evidence for additional candidate genes, including a strong but not exome-wide significant association between *NBN* mutations and the presence of metastases, and a nominal association between *MSH2* mutations and aggressive classification [21]. It is important to note that the majority of samples (85%) included in our study had also been sequenced as part of this larger international effort, although these represented only 33% of the samples in the multicountry study. However, rare pLOF mutation frequencies may vary between European ancestry subpopulations from different countries, and the present analysis focuses on the UK population. A comparison of the results from these two studies therefore demonstrates a consistent association between rare germline *ATM* and *BRCA2* pLOF mutations and aggressive PrCa across European ancestry populations. In the multiancestry study, *NBN* mutations had been reported at higher frequencies in the UK population than in the other European populations, and therefore examining this population exclusively in our study may have reduced diminution of association due to population stratification. In the present study, pLOF *NBN* mutations were found in 13 individuals with aggressive PrCa (0.37%), one with intermediate aggressiveness disease (0.11%), and none were found in patients with nonaggressive PrCa. A Slavic founder mutation in *NBN* (p.Lys219fs, rs587776650) has also previously been reported to associate with PrCa risk and survival [31]. This variant was not included in our analyses according to the definition of rare pLOF variants that we employed due to MAF >0.01 in a reference population; however, it was additionally present in six (0.17%) patients with aggressive PrCa relative to one (0.04%) with nonaggressive disease. In our study, we also observed associations between *MSH2* and both tumour stage and Gleason grade group, and between *MLH1* and nodal spread. These phenotypes had not been examined individually in previous large-scale sequencing studies for PrCa aggressiveness; therefore, in spite of the modest numbers of carriers in our study, our results represent the strongest evidence to date of poorer prognostic outcomes for PrCa patients who are carriers of pLOF mutations in these two Lynch syndrome–linked genes with low population mutation frequencies.

Importantly, we were also able to demonstrate that carriers of pLOF mutations in *ATM*, *BRCA2*, *MLH1*, *MSH2*, and *NBN* experience shorter time to death from PrCa after diagnosis. Conversely, aside from *BRCA2*, no reduction in survival time for PrCa cases dying from other non-PrCa causes was observed between carriers and noncarriers, although this analysis was limited by a lower number of events. Whilst our results suggest that *BRCA2* is the main contributor to the association between being a mutation carrier in these five genes and PrCa-specific survival, carrying a mutation in the remaining four genes was also associated with shorter time to death due to PrCa.

We also observed higher pLOF mutation carrier frequencies with increases in both primary tumour stage and Gleason grade group. A poorer prognosis of PrCa patients with increasing Gleason grade group has previously been established [32,33], although the optimal treatment approaches remain unclear for patients with intermediate-risk disease [34,35], commonly defined as prostate-confined tumours with grade group 2 (Gleason score 3 + 4) or grade group 3 (Gleason score 4 + 3) and PSA levels within a specified range. Further studies to evaluate whether pLOF mutations in the genes associated with aggressiveness could distinguish a subset of individuals presenting with intermediate-risk PrCa at a greater likelihood of progression to aggressive PrCa, or facilitate the selection of patients among this risk group who are more or less likely to be suitable for active surveillance as a treatment option, may therefore be warranted.

Of the five genes for which no examined phenotype met the predefined significance thresholds, we observed higher frequencies of *BRCA1* and *PALB2* pLOF mutations among PrCa cases that had presented with clinical indicators of more aggressive PrCa, suggestive of possible associations with these additional genes, which may warrant further evaluation in larger sample sizes. We found no evidence in support of associations with aggressive disease indicators among *CHEK2* and *MUTYH* mutation carriers despite a relatively high number of carriers of mutations in these genes within our dataset, nor for *MSH6*, for which the carrier frequency was low. Of the 11 men with a pLOF mutation in more than one candidate gene, nine carried *CHEK2* or *MUTYH* variants, for eight of whom this was alongside a mutation of one of the five genes associated with aggressiveness. This further suggests that *CHEK2* and *MUTYH* pLOF variants are less likely to influence PrCa prognosis, although would not preclude their potential association with overall PrCa incidence, as has convincingly been demonstrated for *CHEK2* [8,9,19,36]. Conflicting results as to whether pLOF *CHEK2* mutations additionally predispose towards more aggressive disease had been reported previously [19,37], with our comparatively large single-population study supporting other more recent reports that did not find evidence for *CHEK2* pLOF variants conferring a substantial contribution towards a greater likelihood of aggressive disease in men diagnosed with PrCa [21,36]. A lower effect on overall PrCa risk has also been reported for the *CHEK2* I157T (p.Ile157Thr, rs17879961) missense variant than for protein truncating *CHEK2* variants [38]; however, this variant was not included in our analyses based upon the definition of rare pLOF variants that we employed, and therefore this more abundant but potentially less clinically significant variant was not a potential source of type II error in our analyses. Biallelic pathogenic *MUTYH* mutations substantially increase colorectal cancer risk, but whether risks are also elevated for heterozygote *MUTYH* carriers or predispose towards additional cancer types remains unclear and controversial [39,40], and the association between *MUTYH* mutations and either PrCa onset or aggressiveness has not adequately been studied previously. A recent report identified that heterozygous germline pLOF *MUTYH* mutations were observed twice as frequently in The Cancer Genome Atlas prostate adenocarcinoma cohort than in gnomAD cancer–free individuals [41]; however, population stratification between these disparate cohorts cannot be discounted. We observed a 2.0% carrier frequency for *MUTYH* pLOF mutations among the PrCa cases in this study, in line with the reported heterozygous *MUTYH* pathogenic variant carrier frequencies from other predominantly European ancestry populations [42]. No evidence was found for a greater risk of poorer prognosis disease among *MUTYH* carriers, and we were not able to evaluate whether the *MUTYH* carrier rate was elevated relative to healthy controls from the UK population in our case-only sample, nor examine the effect of biallelic germline *MUTYH* mutations or the occurrence of somatic loss of heterozygosity on the risk of aggressive PrCa.

We also examined whether a GRS derived from common variation and developed to predict PrCa incidence could, in conjunction with information from rare pLOF mutations, provide additional information towards the likelihood of aggressive PrCa classification, as had been reported previously for overall PrCa incidence [43] and potentially also mortality [44]. Opposing directions of association with aggressive status were observed for GRS and mutation status, with GRS modestly associated with nonaggressive classification and being a mutation carrier with aggressiveness, and no interaction between GRS and mutation status. The association between GRS and nonaggressive classification was not significant upon the removal of 51 variants that have also been reported to associate with PSA expression from the GRS, supporting previous observations that accounting for baseline PSA when incorporating information from a GRS into decisions on whether or not to biopsy may have the potential to reduce the likelihood of overdiagnosis of individuals with lower-risk tumours [30].

Whilst our approach of collating data from multiple previous studies to maximise the power available with existing resources enabled us to detect associations with PrCa aggressiveness for five of the candidate genes, our ability to detect moderate strength associations at genes with low pLOF mutation rates within the UK population remained limited in this analysis. Since higher pLOF variant carrier frequencies were observed among poorer prognosis phenotype groups for *BRCA1* and *PALB2*, we are therefore unable to exclude that these genes could also contribute towards aggressive disease. The absence of data from controls in this investigation precluded us from conclusively establishing that the genes in which pLOF mutations demonstrated associations between aggressive and nonaggressive PrCa cases equated to an association specifically with a greater risk of aggressive disease. Nontruncating pathogenic mutations currently classified as variants of uncertain significance (VUS) could also confer greater importance on the genes for which we were not able to establish an association with PrCa prognosis; however, we were unable to examine VUS due to challenges in accurately evaluating the likelihood of pathogenicity for nontruncating mutations for the majority of genes examined. In addition, missing phenotype data within the UKGPCS may have further reduced statistical power in some analyses, with incomplete treatment data rendering us unable to interrogate potential relationships between types of treatment and prognostic outcomes for mutation carriers, as has previously been demonstrated for *BRCA2* mutation carriers [16].

## 5. Conclusions

This study provides support for a role of rare pathogenic germline mutations in the risk of aggressive PrCa. Our findings help to define the panel of genes for which sequencing would be informative for the identification of men at an elevated risk of PrCa with a poorer prognosis, and for treatment decisions for mutation carriers presenting with low- or intermediate-risk disease, especially those diagnosed at younger ages.

## Supplementary Material

Supplementary Figures

Supplementary Tables

## Figures and Tables

**Table 1 –:** Clinical characteristics of the total UK population European ancestry pooled analysis cohort and stratified by aggressiveness classification subgroups

	Total	Aggressive	Intermediate	Nonaggressive
Total PrCa cases (*N*)	6805	3548	898	2359
Age at diagnosis, median (IQR)	58 (55–64)	59 (56–67)	57 (55–59)	58 (55–62)
Follow-up (yr), median (IQR)	10.4 (6.2–15.3)	7.0 (4.0–10.8)	15.2 (12.9–17.1)	13.9 (9.5–17.2)
PSA at diagnosis (ng/ml), median (IQR)	9.5 (5.3–32.8)	26.3 (8.1–92)	8.1 (5.6–14.4)	6.3 (4.5–9.0)
Unknown, *N* (PSA)	435	253	70	112
GRS, median (IQR)	31.4 (30.8–32.1)	31.4 (30.8–32.0)	31.6 (31.0–32.2)	31.5 (30.9–32.1)
Unknown, *N* (GRS)	1199	637	83	479
PrCa family history, *N* (%)				
Yes	2329 (37.4)	1037 (33.2)	437 (50.1)	855 (38.3)
No	3893 (62.6)	2082 (66.8)	436 (49.9)	1375 (61.7)
Unknown	583	429	25	129
Gleason score, *N* (%)				
≤6	3121 (51.5)	417 (14.6)	345 (40.5)	2359 (100.0)
7	1071 (17.7)	627 (22.0)	444 (52.1)	NA
≥8	1867 (30.8)	1804 (63.3)	63 (7.4)	NA
Unknown	746	700	46	NA
Tumour stage, *N* (%)				
T1	2129 (35.2)	186 (6.5)	121 (14.6)	1822 (77.2)
T2	1547 (25.6)	496 (17.4)	514 (61.9)	537 (22.8)
T3	1953 (32.3)	1757 (61.6)	196 (23.6)	NA
T4	414 (6.9)	414 (14.5)	NA	NA
Unknown	762	695	67	NA
Lymph node spread, *N* (%)				
Yes	774 (18.0)	746 (33.8)	19 (3.0)	9 (0.6)
No	3521 (82.0)	1462 (66.2)	617 (97.0)	1442 (99.4)
Unknown	2510	1340	262	908
Metastatic spread, *N* (%)				
Yes	1047 (23.9)	1047 (40.9)	NA	NA
No	3335 (76.1)	1516 (59.1)	581 (100.0)	1238 (100.0)
Unknown	2423	985	317	1121
Cause of death, *N* (%)				
Prostate cancer	2561 (37.6)	2561 (72.2)	NA	NA
Other cause	512 (7.5)	129 (3.6)	90 (10.0)	293 (12.4)
Unknown cause	88 (1.3)	80 (2.3)	3 (0.3)	5 (0.2)
Alive	3644 (53.5)	778 (21.9)	805 (89.6)	2061 (87.4)

GRS = genetic risk score; IQR = interquartile range; NA = not applicable; PrCa = prostate cancer; PSA = prostate-specific antigen.

Variables indicated with NA indicate phenotypes precluded by the criteria used to define the classifications. Samples classified to be of intermediate aggressiveness were not included in the primary analysis, but contribute to the secondary, survival, and GRS analyses.

**Table 2 –:** Survival analysis results for PrCa-specific, all-cause, and non-PrCa mortality by carrier status for the five genes associated with aggressive PrCa (*ATM*, *BRCA2*, *MLH1*, *MSH2*, and *NBN*), and with *BRCA2* separate from the remainder of the gene set

	Censored (*N*)	Deaths (*N*)	Total, *N*	(%)	Person-years of follow-up, *N* (%)		Hazard ratio	95% CI	*p* value
*PrCa-specific mortality, by mutation status in five genes*									
No mutation	4068	2375	6443	(96.5)	52 727	(97.4)	Ref.		
Mutation	91	145	236	(3.5)	1401	(2.6)	2.15	1.79, 2.59	4 × 10^−16^
*All-cause mortality, by mutation status in five genes*									
No mutation	3511	2932	6443	(96.5)	52 727	(97.4)	Ref.		
Mutation	72	164	236	(3.5)	1401	(2.6)	2.15	1.84, 2.52	2 × 10^−21^
*Non-PrCa mortality, by mutation status in five genes*									
No mutation	5886	557	6443	(96.5)	52 727	(97.4)	Ref.		
Mutation	217	19	236	(3.5)	1401	(2.6)	1.17	0.74, 1.85	0.5
*PrCa-specific mortality, by mutation status in four genes (minus BRCA2)*									
No mutation	4103	2450	6553	(98.1)	53 271	(98.4)	Ref.		
Mutation	56	70	126	(1.9)	857	(1.6)	1.78	1.38, 2.30	9 × 10^−6^
*All-cause mortality, by mutation status in four genes (minus BRCA2)*									
No mutation	3534	3019	6553	(98.1)	53 271	(98.4)	Ref.		
Mutation	49	77	126	(1.9)	857	(1.6)	1.59	1.27, 2.00	5 × 10^−5^
*Non-PrCa mortality, by mutation status in four genes (minus BRCA2)*									
No mutation	5984	569	6553	(98.1)	53 271	(98.4)	Ref.		
Mutation	119	7	126	(1.9)	857	(1.6)	0.73	0.36, 1.50	0.4
*PrCa-specific mortality, by mutation status in BRCA2*									
No mutation	4123	2444	6567	(98.3)	53 573	(99.0)	Ref.		
Mutation	36	76	112	(1.7)	555	(1.0)	2.56	1.98, 3.31	1 × 10^−12^
*All-cause mortality, by mutation status in BRCA2*									
No mutation	3559	3008	6567	(98.3)	53 573	(99.0)	Ref.		
Mutation	24	88	112	(1.7)	555	(1.0)	2.96	(2.39, 3.66)	2 × 10^−23^
*Non-PrCa mortality, by mutation status in BRCA2*									
No mutation	6003	564	6567	(98.3)	53 573	(99.0)	Ref.		
Mutation	100	12	112	(1.7)	555	(1.0)	1.75	(0.97, 3.17)	0.06

CI = confidence interval; PrCa = prostate cancer; Ref. = reference.

## References

[1] International Agency for Research on Cancer. World Health Organization. Global Cancer Observatory https://gco.iarc.fr/today/online-analysis-table.

[2] Sung H, Ferlay J, Siegel RL, Global cancer statistics 2020: GLOBOCAN estimates of incidence and mortality worldwide for 36 cancers in 185 countries. CA Cancer J Clin 2021;71:209–49. 33538338 10.3322/caac.21660

[3] Howlader N, Noone AM, Krapcho M, SEER cancer statistics review, 1975–2017. Bethesda, MD: National Cancer Institute. https://seer.cancer.gov/csr/1975_2017/.

[4] Siegel RL, Miller KD, Jemal A. Cancer statistics, 2020. CA Cancer J Clin 2020;70:7–30. 31912902 10.3322/caac.21590

[5] Hjelmborg JB, Scheike T, Holst K, The heritability of prostate cancer in the Nordic Twin Study of Cancer. Cancer Epidemiol Biomarkers Prev 2014;23:2303–10. 24812039 10.1158/1055-9965.EPI-13-0568 PMC4221420

[6] Lichtenstein P, Holm NV, Verkasalo PK, Environmental and heritable factors in the causation of cancer—analyses of cohorts of twins from Sweden, Denmark, and Finland. N Engl J Med 2000;343:78–85. 10891514 10.1056/NEJM200007133430201

[7] Saunders EJ, Kote-Jarai Z, Eeles RA. Identification of germline genetic variants that increase prostate cancer risk and influence development of aggressive disease. Cancers (Basel) 2021;13:760. 33673083 10.3390/cancers13040760 PMC7917798

[8] Conti DV, Darst BF, Moss LC, Trans-ancestry genome-wide association meta-analysis of prostate cancer identifies new susceptibility loci and informs genetic risk prediction. Nat Genet 2021;53:65–75. 33398198 10.1038/s41588-020-00748-0 PMC8148035

[9] Wang A, Shen J, Rodriguez AA, Characterizing prostate cancer risk through multi-ancestry genome-wide discovery of 187 novel risk variants. Nat Genet 2023;55:2065–74. 37945903 10.1038/s41588-023-01534-4 PMC10841479

[10] Green HD, Merriel SWD, Oram RA, Applying a genetic risk score for prostate cancer to men with lower urinary tract symptoms in primary care to predict prostate cancer diagnosis: a cohort study in the UK Biobank. Br J Cancer 2022;127:1534–9. 35978138 10.1038/s41416-022-01918-z PMC9553867

[11] Klein RJ, Vertosick E, Sjoberg D, Prostate cancer polygenic risk score and prediction of lethal prostate cancer. NPJ Precis Oncol 2022;6:25. 35396534 10.1038/s41698-022-00266-8 PMC8993880

[12] Ma C, Ericsson C, Carlsson SV, Addition of a genetic risk score for identification of men with a low prostate-specific antigen level in midlife at risk of developing lethal prostate cancer. Eur Urol Open Sci 2023;50:27–30. 36861107 10.1016/j.euros.2023.01.012 PMC9969275

[13] Ewing CM, Ray AM, Lange EM, Germline mutations in HOXB13 and prostate-cancer risk. N Engl J Med 2012;366:141–9. 22236224 10.1056/NEJMoa1110000 PMC3779870

[14] Kote-Jarai Z, Mikropoulos C, Leongamornlert DA, Prevalence of the HOXB13 G84E germline mutation in British men and correlation with prostate cancer risk, tumour characteristics and clinical outcomes. Ann Oncol 2015;26:756–61. 25595936 10.1093/annonc/mdv004

[15] Xu J, Lange EM, Lu L, HOXB13 is a susceptibility gene for prostate cancer: results from the International Consortium for Prostate Cancer Genetics (ICPCG). Hum Genet 2013;132:5–14. 23064873 10.1007/s00439-012-1229-4 PMC3535370

[16] Castro E, Goh C, Leongamornlert D, Effect of BRCA mutations on metastatic relapse and cause-specific survival after radical treatment for localised prostate cancer. Eur Urol 2015;68:186–93. 25454609 10.1016/j.eururo.2014.10.022

[17] Castro E, Goh C, Olmos D, Germline BRCA mutations are associated with higher risk of nodal involvement, distant metastasis, and poor survival outcomes in prostate cancer. J Clin Oncol 2013;31:1748–57. 23569316 10.1200/JCO.2012.43.1882 PMC3641696

[18] Darst BF, Dadaev T, Saunders E, Germline sequencing DNA repair genes in 5,545 men with aggressive and non-aggressive prostate cancer. J Natl Cancer Inst 2021;113:616–25. 32853339 10.1093/jnci/djaa132 PMC8599772

[19] Leongamornlert DA, Saunders EJ, Wakerell S, Germline DNA repair gene mutations in young-onset prostate cancer cases in the UK: evidence for a more extensive genetic panel. Eur Urol 2019;76:329–37. 30777372 10.1016/j.eururo.2019.01.050 PMC6695475

[20] Na R, Zheng SL, Han M, Germline mutations in ATM and BRCA1/2 distinguish risk for lethal and indolent prostate cancer and are associated with early age at death. Eur Urol 2017;71:740–7. 27989354 10.1016/j.eururo.2016.11.033 PMC5535082

[21] Darst BF, Saunders E, Dadaev T, Germline sequencing analysis to inform clinical gene panel testing for aggressive prostate cancer. JAMA Oncol 2023;9:1514–24. 37733366 10.1001/jamaoncol.2023.3482 PMC10881219

[22] Cybulski C, Wokolorczyk D, Kluzniak W, An inherited NBN mutation is associated with poor prognosis prostate cancer. Br J Cancer 2013;108:461–8. 23149842 10.1038/bjc.2012.486 PMC3566821

[23] Brook MN, Ni Raghallaigh H, Govindasami K, Family history of prostate cancer and survival outcomes in the UK Genetic Prostate Cancer Study. Eur Urol 2023;83:257–66. 36528478 10.1016/j.eururo.2022.11.019

[24] Leongamornlert D, Saunders E, Dadaev T, Frequent germline deleterious mutations in DNA repair genes in familial prostate cancer cases are associated with advanced disease. Br J Cancer 2014;110:1663–72. 24556621 10.1038/bjc.2014.30 PMC3960610

[25] Mijuskovic M, Saunders EJ, Leongamornlert DA, Rare germline variants in DNA repair genes and the angiogenesis pathway predispose prostate cancer patients to develop metastatic disease. Br J Cancer 2018;119:96–104. 29915322 10.1038/s41416-018-0141-7 PMC6035259

[26] Schaid DJ, McDonnell SK, FitzGerald LM, Two-stage study of familial prostate cancer by whole-exome sequencing and custom capture identifies 10 novel genes associated with the risk of prostate cancer. Eur Urol 2021;79:353–61. 32800727 10.1016/j.eururo.2020.07.038 PMC7881048

[27] McLaren W, Gil L, Hunt SE, The Ensembl variant effect predictor. Genome Biol 2016;17:122. 27268795 10.1186/s13059-016-0974-4 PMC4893825

[28] Landrum MJ, Lee JM, Benson M, ClinVar: improving access to variant interpretations and supporting evidence. Nucleic Acids Res 2018;46:D1062–7. 29165669 10.1093/nar/gkx1153 PMC5753237

[29] Schumacher FR, Al Olama AA, Berndt SI, Association analyses of more than 140,000 men identify 63 new prostate cancer susceptibility loci. Nat Genet 2018;50:928–36. 29892016 10.1038/s41588-018-0142-8 PMC6568012

[30] Kachuri L, Hoffmann TJ, Jiang Y, Genetically adjusted PSA levels for prostate cancer screening. Nat Med 2023;29:1412–23. 37264206 10.1038/s41591-023-02277-9 PMC10287565

[31] Rusak B, Kluzniak W, Wokolorczykv D, Inherited NBN mutations and prostate cancer risk and survival. Cancer Res Treat 2019;51:1180–7. 30590007 10.4143/crt.2018.532 PMC6639207

[32] Epstein JI, Zelefsky MJ, Sjoberg DD, A contemporary prostate cancer grading system: a validated alternative to the Gleason score. Eur Urol 2016;69:428–35. 26166626 10.1016/j.eururo.2015.06.046 PMC5002992

[33] Pierorazio PM, Walsh PC, Partin AW, Epstein JI. Prognostic Gleason grade grouping: data based on the modified Gleason scoring system. BJU Int 2013;111:753–60. 23464824 10.1111/j.1464-410X.2012.11611.x PMC3978145

[34] Blas L, Shiota M, Eto M. Active surveillance in intermediate-risk prostate cancer: a review of the current data. Cancers (Basel) 2022;14:4161. 36077698 10.3390/cancers14174161 PMC9454661

[35] Courtney PT, Deka R, Kotha NV, Metastasis and mortality in men with low- and intermediate-risk prostate cancer on active surveillance. J Natl Compr Canc Netw 2022;20:151–9. 35130495 10.6004/jnccn.2021.7065 PMC10399925

[36] Wokolorczyk D, Kluzniak W, Huzarski T, Mutations in ATM, NBN and BRCA2 predispose to aggressive prostate cancer in Poland. Int J Cancer 2020;147:2793–800. 32875559 10.1002/ijc.33272

[37] Wu Y, Yu H, Zheng SL, A comprehensive evaluation of CHEK2 germline mutations in men with prostate cancer. Prostate 2018;78:607–15. 29520813 10.1002/pros.23505

[38] Cybulski C, Huzarski T, Gorski B, A novel founder CHEK2 mutation is associated with increased prostate cancer risk. Cancer Res 2004;64:2677–9. 15087378 10.1158/0008-5472.can-04-0341

[39] Curia MC, Catalano T, Aceto GM. MUTYH: not just polyposis. World J Clin Oncol 2020;11:428–49. 32821650 10.5306/wjco.v11.i7.428 PMC7407923

[40] Magrin L, Fanale D, Brando C, MUTYH-associated tumor syndrome: the other face of MAP. Oncogene 2022;41:2531–9. 35422474 10.1038/s41388-022-02304-y

[41] Barreiro RAS, Sabbaga J, Rossi BM, Monoallelic deleterious MUTYH germline variants as a driver for tumorigenesis. J Pathol 2022;256:214–22. 34816434 10.1002/path.5829

[42] Win AK, Jenkins MA, Dowty JG, Prevalence and penetrance of major genes and polygenes for colorectal cancer. Cancer Epidemiol Biomarkers Prev 2017;26:404–12. 27799157 10.1158/1055-9965.EPI-16-0693 PMC5336409

[43] Darst BF, Sheng X, Eeles RA, Kote-Jarai Z, Conti DV, Haiman CA. Combined effect of a polygenic risk score and rare genetic variants on prostate cancer risk. Eur Urol 2021;80:134–8. 33941403 10.1016/j.eururo.2021.04.013 PMC8286329

[44] Shi Z, Platz EA, Wei J, Performance of three inherited risk measures for predicting prostate cancer incidence and mortality: a population-based prospective analysis. Eur Urol 2021;79:419–26. 33257031 10.1016/j.eururo.2020.11.014

