# Supplementary material for: Identification of Genes with Rare Loss of Function Variants Associated with Aggressive Prostate Cancer and Survival
Source: Eur Urol Oncol. Author manuscript; Available in PMC 2025 May 30. (PMC12124132; doi:10.1016/j.euo.2024.02.003)
Supplement: Supplementary Figures [file NIHMS2085376-supplement-Supplementary_Figures.pdf]

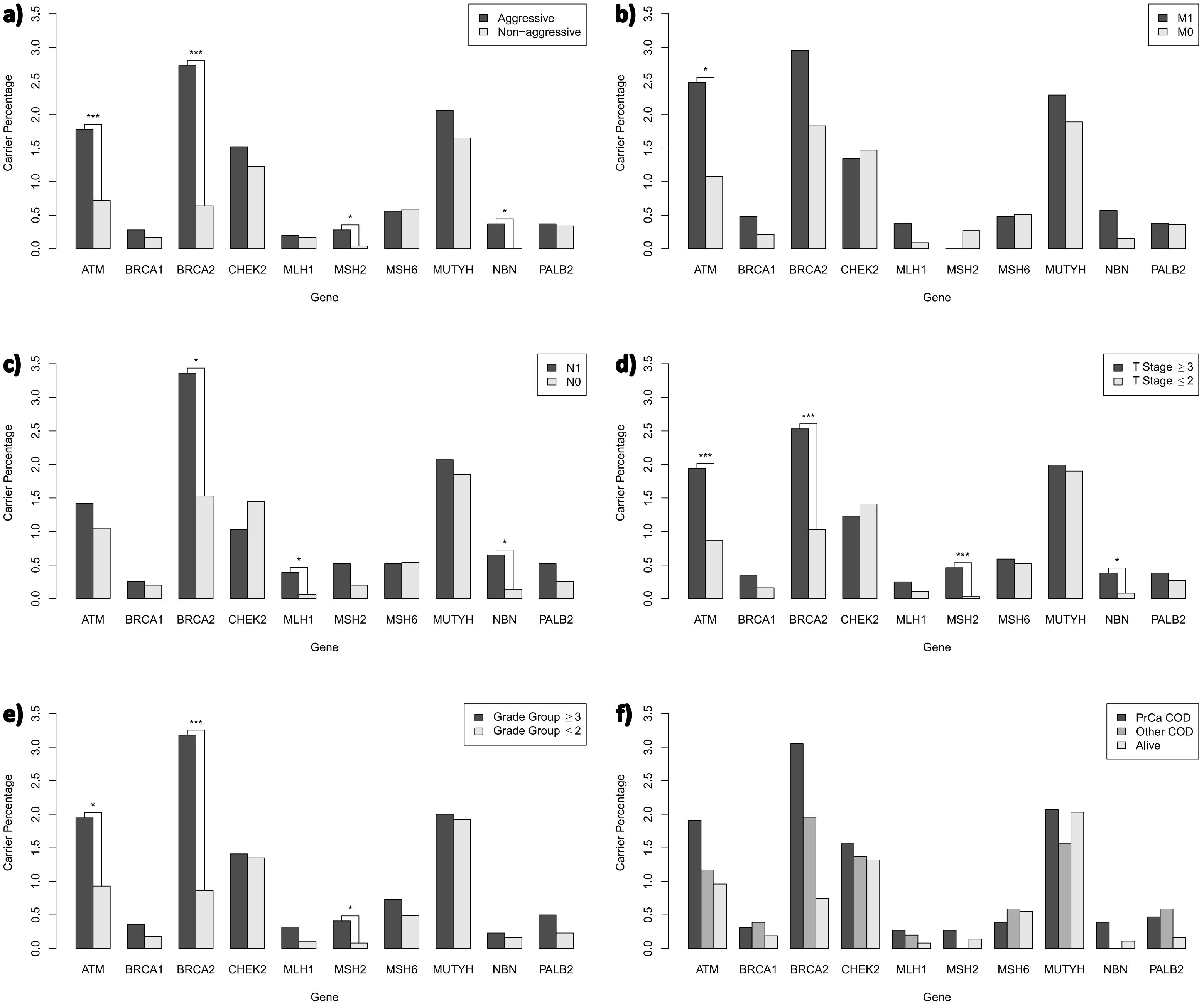

Supplementary Figure 1 – Relative pLOF mutation carrier frequencies between PrCa phenotypes for the ten genes examined in the meta-analysis dataset. a) Aggressive classification, **b)** Presence (M1) or absence (M0) of distant metastases, **c)** Presence (N1) or absence (N0) of nodal spread, **d)** Higher or lower primary tumour stage **e)** Higher or lower Gleason grade group and **f)** Cause of death (COD). Genes associated at *P*<0.001 are indicated with *******, and at *P*<0.05 with *****.

a)

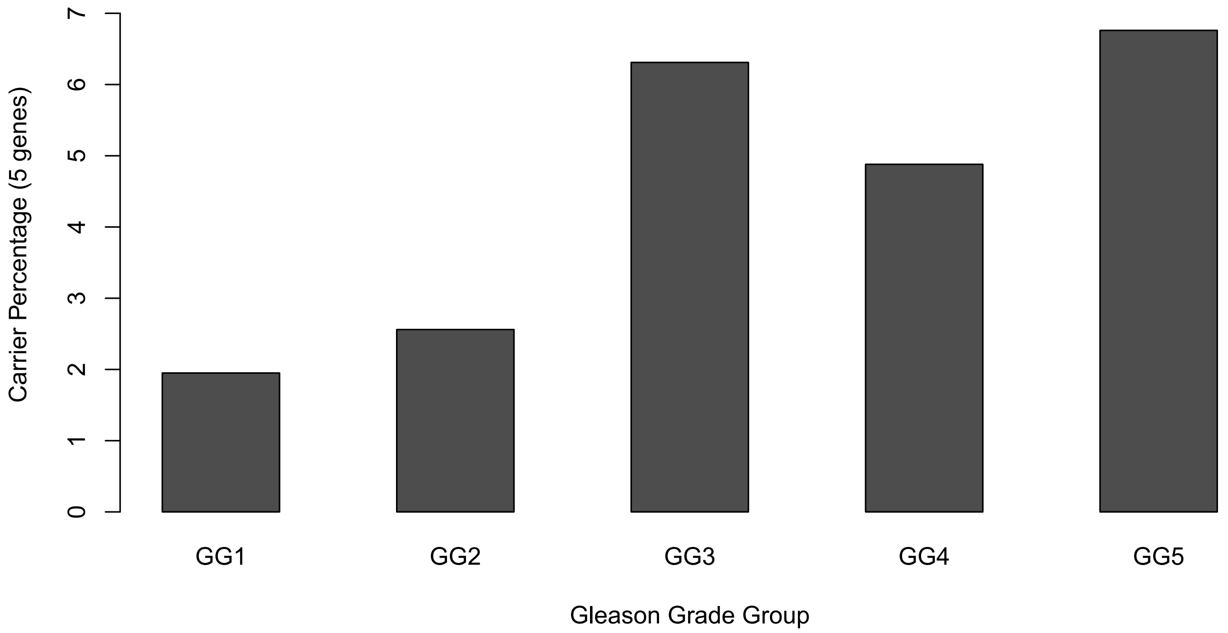

b)

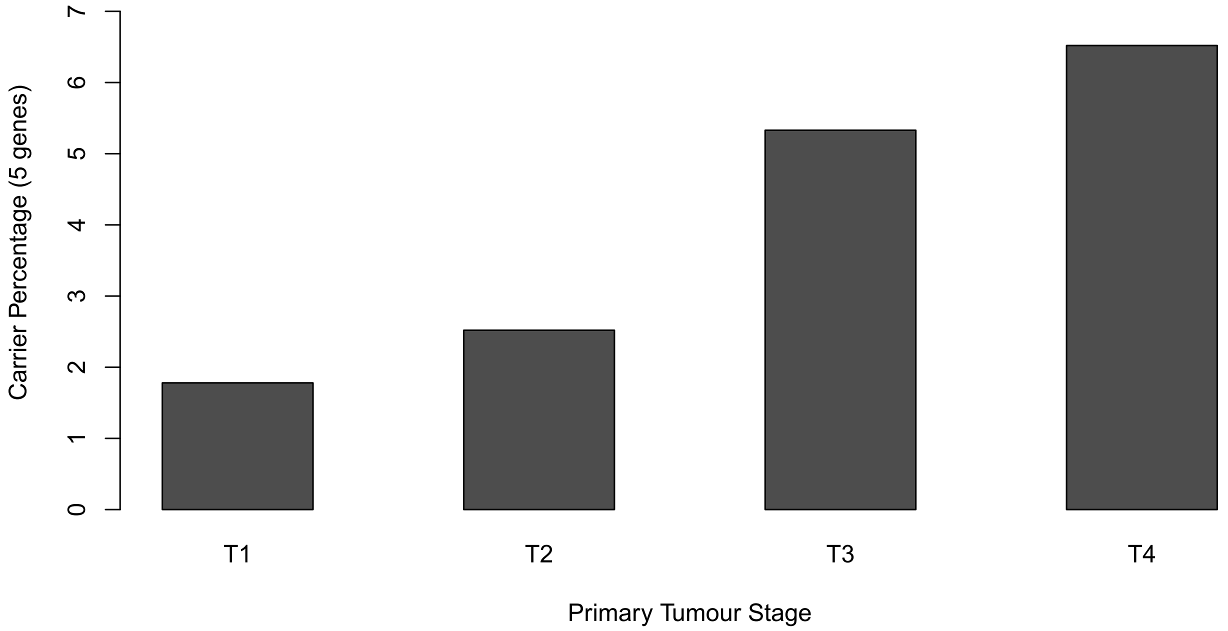

Supplementary Figure 2 – Relative pLOF mutation carrier frequencies for the five genes associated with aggressive PrCa (*ATM*, *BRCA2*, *MLH1*, *MSH2* and *NBN*) between different primary tumour phenotype category groupings. **a)** Gleason grade groups, and **b)** Tumour Stage.
